# Electron-Optical In Situ Imaging for the Assessment of Accuracy in Electron Beam Powder Bed Fusion

**DOI:** 10.3390/ma14237240

**Published:** 2021-11-26

**Authors:** Christopher Arnold, Christoph Breuning, Carolin Körner

**Affiliations:** Department of Materials Science and Engineering, Chair of Materials Science and Engineering for Metals, Friedrich-Alexander-Universität Erlangen-Nürnberg (FAU), Martensstr. 5, 91058 Erlangen, Germany; christoph.breuning@fau.de (C.B.); carolin.koerner@fau.de (C.K.)

**Keywords:** additive manufacturing, electron beam, powder bed fusion, electron imaging, in situ measurement, process monitoring, computed tomography, thermodynamic simulation, geometrical defects, surface roughness

## Abstract

The current study evaluates the capabilities of electron-optical (ELO) in situ imaging with respect to monitoring and prediction of manufacturing precision in electron beam powder bed fusion. Post-process X-ray computed tomography of two different as-built parts is used to quantitatively evaluate the accuracy and limitations of ELO imaging. Additionally, a thermodynamic simulation is performed to improve the understanding of ELO data and to assess the feasibility of predicting dimensional accuracy numerically. It is demonstrated that ELO imaging captures the molten layers accurately (deviations <100 μm) and indicates the creation of surface roughness. However, some geometrical features of the as-built parts exhibit local inaccuracies associated with thermal stress-induced deformation (deviations up to 500 μm) which cannot be captured by ELO imaging. It is shown that the comparison between in situ and post-process data enables a quantification of these effects which might provide the possibility for developing effective countermeasures in the future.

## 1. Introduction

Electron beam powder bed fusion (PBF-EB) is a metal additive manufacturing (AM) process that enables the tool-free production of complex shaped parts. Despite this great asset, the application of AM technologies in industrial production is still hindered by an inferior geometrical and dimensional accuracy compared to conventional manufacturing technologies. To overcome these limitations, many efforts are put into the further development of metrology and specification standards dedicated to the specific needs of metal AM [1]. The accuracy of AM processes is usually assessed by manufacturing of benchmark artifacts [1,2,3] with standardized and differently sized geometrical features. For metal powder bed fusion, Gruber et al. [4] showed that the obtained accuracy of the manufactured artifacts could be related to basic process characteristics like beam diameter and layer height. However, also the feedstock material and the quality of the corresponding process parameters strongly affected the results [4]. The strong influence of process parameters has also been shown by Smith et al. [5] for the dimensional accuracy of truss structures manufactured by PBF-EB. Another important factor that up to now has hardly been considered when discussing geometrical and dimensional accuracy is the effect of the chosen scanning strategy, especially for the manufacturing of complex shaped parts. It has already been shown that the scanning strategy is a powerful tool to tailor the energy input and thereby controlling microstructure and properties of the manufactured parts by PBF-EB [6,7]. However, most of the machines in the field only provide limited control over the scanning strategy to the operator. This circumstance impedes the absolute assessment of an AM technology as a whole based on manufacturing of an arbitrary geometry, e.g., a benchmark artifact. Different studies have shown that the geometric errors produced by PBF-EB exhibit a high level of repeatability [8,9]. Thus, advanced machine control and a deeper understanding of the physical processes involved are necessary to develop effective countermeasures and to reveal the actual capabilities of metal AM with respect to geometrical and dimensional accuracy.

One approach to gain more insights into the manufacturing process is the application of simulations. Since the occurrence of thermal stresses was identified to be the main cause of severe part distortion, several thermo-mechanical frameworks were developed to simulate the manufacturing process on different levels of detail [10]. Only a few of these investigations were dedicated to the specific characteristics of PBF-EB while most of them targeted laser powder bed fusion (PBF-LB). For the electron beam process, large-scale simulations based on finite element analysis (FEA) [11] or the inherent strain method (IS) [12] were conducted to simulate the macroscopic warping of complete build jobs. However, these methods were not capable of resolving the small-scale distortion of single layers or parts. Cheng and Chou [13] applied a much more detailed FEA model to simulate the warping in overhang regions for a small number of layers [13] and the effect of an underlying support structure [14,15]. Ghaoui et al. [16] developed a model to simulate the edge loss effect and achieved a good agreement with experimental data. In total, these more detailed simulations provided the opportunity to validate theoretical models for simple and small-scale geometries. However, due to the high computational costs involved, the presented models were not yet used to predict distortion of large-scale and complex-shaped parts.

Another approach to gather novel information is in situ measurement of process characteristics. A large variety of methods operating at different time and length scales was investigated in recent years. A general overview of this dynamic field of research may be obtained from comprehensive review papers [17,18,19,20]. Within this context, in situ assessment of geometrical and dimensional accuracy of the molten layers is only one aspect. Again, most of the conducted investigations have been performed for PBF-LB where mainly visible light imaging was used to acquire layer-wise images of the molten surfaces. These images were then used to extract the geometry information [21] and perform comparisons with references obtained from computer aided design (CAD) and post-process X-ray computed tomography (XCT) [22]. Due to the complex interaction of surface structure and illumination conditions, a robust and reliable segmentation of the molten contours is a major challenge of this approach. Therefore, a large variety of advanced segmentation algorithms was developed and tested for different camera set-ups and illumination conditions [23,24,25,26,27,28,29]. In PBF-EB, due to the high processing temperature and the associated incandescence of the build surface, imaging in the visible range is hardly applied. Most investigations deal with infrared (IR) imaging [20] which also contains information on surface temperature [30,31]. Price et al. [32] applied a near infrared (NIR) camera to measure the decreased cooling rate in an overhang region which is supposed to affect thermal stresses and thus distortion. Ridwan et al. [33] were the first to directly evaluate the accuracy of the molten surfaces by comparing IR images to the CAD data of the corresponding layer. Croset et al. [34] used a similar approach with NIR images to detect an excessive energy input by evaluating the overshooting of the melt in the contour region of a molten surface. Recently, Arnold and Körner [35] presented a different approach for assessing the geometrical and dimensional accuracy which is based on the evaluation of electron-optical (ELO) images. The ELO data was first calibrated and then a validation experiment was performed which demonstrated a high agreement with XCT measurement of the as-built part [35].

The current investigation intends to extend this evaluation of ELO imaging data by analyzing the complete three-dimensional information obtained from two different geometries. The data is again compared to the geometry of the as-built parts obtained from XCT measurement to further discuss characteristics and limitations of ELO imaging. An additional focus is put on the influence of melt pool characteristics on the dimensional accuracy and surface roughness of the manufactured parts. For this purpose, simulation of the melt pool extension during manufacturing and the resulting accuracy of the molten layers are incorporated into the investigation and discussed along with ELO and XCT data.

## 2. Materials and Methods

The experimental part of the current study involved a variety of methods for manufacturing, characterization and simulation of the PBF-EB parts. The following sections give the details about acquisition and processing of the corresponding data.

### 2.1. PBF-EB Process and Part Design

Additive manufacturing of the parts was performed using the in-house development PBF-EB system ATHENE. The installed electron beam gun by pro-beam GmbH & Co. KGaA (Gilching, Germany) provides an acceleration voltage of 60 kV and a beam power up to 6 kW. Its electro-magnetic beam deflection system is designed to provide deflection speeds of more than 1000 m/s. Its interface enables the implementation of arbitrary beam paths and scan patterns. Manufacturing of the parts was achieved by layer-wise repetition of the PBF-EB build cycle. First, a recoater system was used for application of a powder layer with a nominal thickness of 50 μm. The feedstock material was plasma atomized Ti-6Al-4V ELI powder with a particle size fraction of 45 μm–105 μm. In the second step, the defocused beam was scanned with high deflection speed across the build surface to preheat the powder and to maintain a processing temperature of around 740 °C. The third step was selective melting of the parts’ current slice cross-section with a high focus, a beam current of 6.7 mA and a deflection speed of 2.67 m/s. To consolidate the layer, a standard cross-snake hatching strategy with a hatch line spacing of 100 μm was applied. The hatching direction was rotated by 90° between layers to homogenize the energy input and avoid material transport effects. The cross-sections of different parts were molten independently. After melting, the build platform was lowered by the height of a single layer and another run of the build cycle was conducted until the part was finished. The entire manufacturing process was performed in a controlled vacuum atmosphere of $3\times 10^{-3}$ mbar helium pressure.

**Figure 1:**
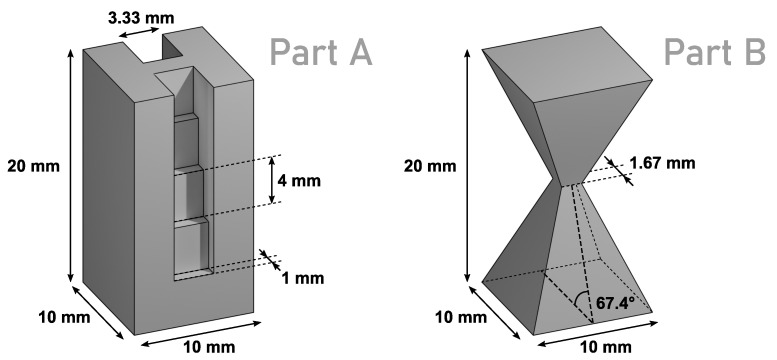
Geometry and dimensions of part A and part B.

The geometries of the two parts which were built for the current investigation are depicted in Figure 1. Part A was a cuboid-shaped specimen with a base-area of 10×10 mm^2^ and a height of 20 mm. A staircase structure with a step height of 4 mm, a step width of 3.33 mm and a step depth of 1 mm was cut from two sides of the cuboid to obtain varying layer cross-sections shaped like the letter “H”. This geometry has already been used by the authors in another investigation to validate the concept of layer-wise electron-optical in situ metrology for PBF-EB [35]. Part B was composed of a right square pyramid with a base of 10×10 mm^2^ and an identical pyramid rotated upside down on top of it. Both pyramids have a theoretical height of 12 mm but their apexes are shifted into each other by 2 mm to obtain a single specimen with a total height of 20 mm. The minimum square cross-section of this geometry is 1.67×1.67 mm^2^ and the dihedral angle between base and side planes is 67.4°. Both parts were connected by a columnar support structure with a height of 5 mm and spacing of 2.5 mm to the underlying steel base plate.

### 2.2. ELO Imaging

To monitor the quality of the molten layers, an additional ELO imaging step was introduced to the PBF-EB build cycle after melting of the current slice cross-section. During ELO imaging the electron beam scanned an exposure area of 70×70 mm^2^ with a low beam current of 3 mA. The approach uses the effect that a fraction of the incident beam electrons is always reflected from the build surface through elastic scattering with the atom cores of the metal feedstock material. These backscattered electrons (BSE) were (partially) recorded using an annular copper plate sensor which was located above the build surface in coaxial position to the optical axis of the electron beam. The BSE sensor and the accompanying signal processing system were designed by pro-beam GmbH & Co. KGaA (Gilching, Germany) for applications in electron beam welding. As the BSE signal strength locally depends on material and topography of the scanned surface, it may be used to generate a BSE intensity map as also known from scanning electron microscopy. The signal was mapped to quadratic ELO images with a size of 1500×1500 px, i.e., an instantaneous field-of-view of 47 μm/px. To increase the accuracy of the subsequent comparison with XCT, the ELO images were post-processed by correction of imaging errors and by virtually including thermal shrinkage occurring during cooling down from processing temperature to room temperature. Further details on these operations are given elsewhere [35]. To obtain a quasi-3D representation of the manufactured parts, the ELO images of all layers were afterwards virtually stacked upon each other to obtain a three-dimensional voxel volume. A marching cubes algorithm [36] was then applied to this voxel volume to approximate the 3D surface of the manufactured part. The implementation of the algorithm was provided by the Python package scikit-image [37]. The global threshold value which is required to set the interface between molten material and sintered powder-bed was chosen based on the gray value distribution of the ELO images. The gray level histogram of all pixels showed a clear bimodal distribution with two distinct peaks representing the two relevant material states. The threshold was set in the middle between those two peaks, also denoted as ISO-50% threshold. The surface information returned by the marching cubes algorithm was converted to the Standard Triangle Language (STL) file format for further processing.

### 2.3. XCT Measurement

To validate the accuracy returned by evaluation of the in situ data, the as-built samples were measured externally using X-ray computed tomography (XCT). The *v|tome|x m 300* XCT system by GE Sensing and Inspection Technologies GmbH (Wunstorf, Germany) was set to generate X-rays with an acceleration voltage of 190 kV and a current of 60 μA. Each part was rotated around its *z*-axis to generate 2000 X-ray projection images which were then used to reconstruct the three-dimensional density distribution with a voxel size of 10.00 μm. The voxel data was further processed using the software suite *VGSTUDIO MAX* by Volume Graphics GmbH (Heidelberg, Germany). First, the volume data was registered by adjusting position and rotation of the part to target values of the CAD model. Then again, a marching cubes algorithm was applied to extract the surface information of each part. In case of the XCT data, the global threshold value required by the algorithm was set to 66.67% between the peaks of the bimodal gray level distribution since the ISO-50% threshold value returned a visual overestimation of the solid volume. The returned surface data was also stored in the STL file format.

### 2.4. Simulation

To simulate the 3D surface of the manufactured parts, the evolution of the temperature field and the emerging meltpool geometries were calculated for each individual layer of the geometry. For this purpose an explicit finite difference (FD) method was implemented in an in-house simulation framework to solve the heat equation in three dimensions,

$$\frac{\partial T}{\partial t}=\nabla \;\cdot \;\left(\alpha \nabla T\right)+\dot{Q}$$

where *T* is the temperature, $\dot{Q}$ is the heat flux of the electron beam heat source and α is the thermal diffusivity. The heat source term $\dot{Q}=\eta \;\cdot \;I\left(x,y\right)\;\cdot \;P$ is modeled as a surface heat flux with a Gaussian distribution of beam intensity *I* at the location (x,y) which is calculated according to,

$$I\left(x,y\right)=\frac{1}{2\pi \sigma^{2}}e^{\frac{-1}{2\pi \sigma^{2}}\left({\left(x-x_{b}\right)}^{2}+{\left(y-y_{b}\right)}^{2}\right)}$$

where $x_{b}$ and $y_{b}$ defines the location of the center of the beam [38]. The variance σ of the distribution is considered as $\sigma =\frac{1}{4}d_{B}$ with the diameter of the electron beam $d_{B}$. The beam power *P* is controlled by the absorption coefficient of electrons η. The model operates on a cubic lattice with constant lattice spacing dx in all directions and with a constant time step dt. It neglects effects of fluid convection, latent heat release, radiation and vaporization. The powder surrounding previously consolidated areas is assumed as a continuum with a thermal diffusivity of an order-of magnitude lower than the solid phase material [39]. The estimated material parameters adapted from Rausch et al. [40] and Smith et al. [39] are summarized in Table 1.

The interface between solid material and powder is solved using harmonic averaging of material properties [41]. The material properties of a lattice cell are changed from powder to solid material upon exceeding the liquidus temperature. Preheating temperature $T_{p}$ boundary conditions are applied at the edges of the powder bed domain, which is large enough so that the boundary conditions do not influence the temperature field of the build.

The simulation domain is initialized with a solid base plate for each geometry. Each subsequent layer is treated individually by addition of powder layers with a thickness of 50 µm on top of the previously calculated layers and is initialized with a constant preheating temperature $T_{p}$. The melted cells of each individual layer are then combined to form a three-dimensional voxel volume. Similar to processing of the ELO data, the volumetric thermal shrinkage of the part during cooling down from processing temperature to room temperature was considered in the simulation data by adjustment of the voxel size. A marching cubes algorithm was applied to the voxel volume to approximate the 3D surface of the simulated part. Again, the returned surface data was stored in the STL file format for further processing.

### 2.5. Data Analysis and Comparison

The data was processed using the open-source software CloudCompare [42]. CloudCompare enables editing and processing of 3D point clouds and triangular meshes. It was used to read the STL files containing the 3D surface information obtained from ELO imaging, XCT measurement and numerical simulation. To compare the accuracy of the models with respect to the CAD reference, the distance computation functionality of CloudCompare was used. By performing a cloud-to-mesh comparison, the signed distance between each vertex of the model objects to the surface of the CAD model was calculated. This approach was favored over the calculation and comparison of the molten area or volume as performed in other investigations [33,34] since it returns additional and more reliable spatial information about accuracy. The 3D visualization of the computed distances was also achieved using CloudCompare. Furthermore, to perform quantitative comparisons between the 3D models, the software was used to sample 10^6^ data points on the surface of each mesh. The obtained signed distance scalar field was further analyzed by calculating the value distributions for histogram comparisons.

## 3. Results

**Figure 2:**
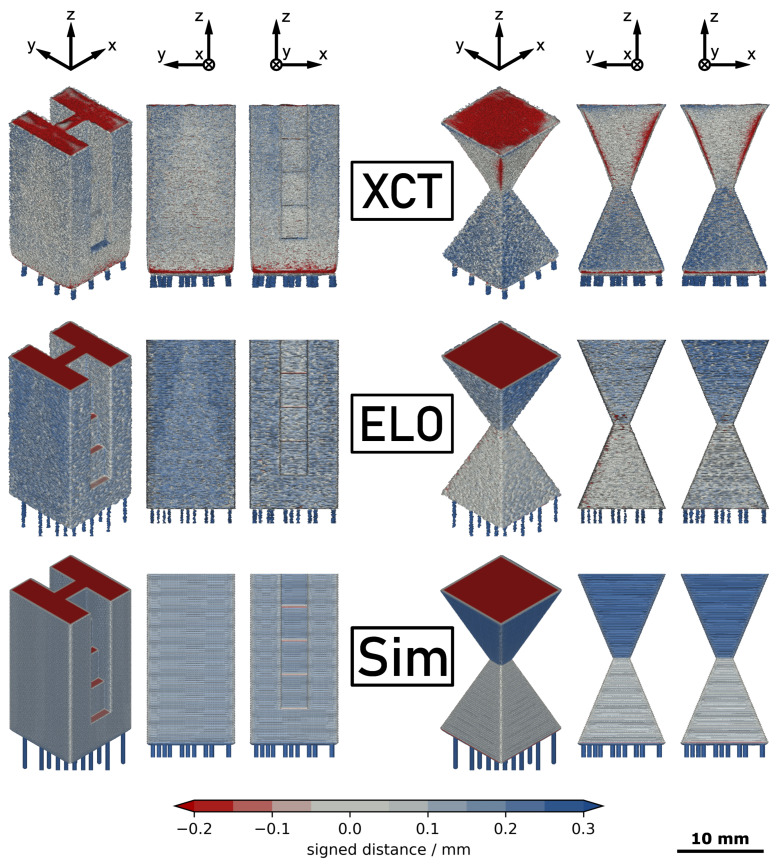
Accuracy of 3D geometry data obtained from X-ray computed tomography (XCT), electron-optical (ELO) imaging and simulation (Sim) with respect to the geometry of the computer aided design (CAD) model for part A (left) and part B (right). Excess and missing material is indicated by blue and red color, respectively.

Figure 2 shows the 3D models returned by XCT measurement, ELO image analysis and thermodynamic simulation. The surface of the models is selectively colored according to the results of the comparison with the CAD model. The color map is composed of three linear segments to indicate the signed distances returned by the calculation. Regions with deviations in the range of ±50 μm are assumed to be in an acceptable manufacturing tolerance and thus are neutrally colored in gray. Distances above +50 μm refer to excess material and are colored blue. Distances below −50 μm indicate missing material and are colored red. The intensity of the colors was linearly increased with larger distance until critical values of +300 μm and −200 μm for excess and missing material, respectively. The lower absolute value for the missing material was chosen due to its more severe implications on post-processing and performance of the manufactured parts. For both parts, the support structure was not included into the CAD reference and thus is also colored as excess material.

### 3.1. XCT Measurement

The upper section of Figure 2 shows the results of the XCT measurement for both parts. The side surfaces of part A exhibit a medium amount of excess material which seems to increase from the bottom to the top of the sample. The quality of the side surfaces of part B clearly depends on the build angle. The upskin surfaces in the lower half of the sample indicate a large amount of excess material. The downskin surfaces in the upper half are mostly closer to the CAD reference but exhibit missing material at the side edges of the pyramidal structure. A similar effect is found at the bottom sides of both part A and part B which also are downskin surfaces. The corresponding edges are strongly rounded, leading to a significant amount of missing material. Both geometries show missing material at their top sides due to the thermal shrinkage after manufacturing of the samples.

### 3.2. ELO Reconstruction

The middle section of Figure 2 displays the result of the 3D reconstruction from in situ ELO images. For part A, a large amount of excess material can be found on the side surfaces. For part B, again it must be distinguished between upskin and downskin surfaces. The upskin surfaces in the lower section are close to the CAD reference with only a small amount of excess material. In contrast, the downskin surfaces of the upper section exhibit a larger amount of excess material, comparable to the side surfaces of part A. As thermal shrinkage of the parts was also considered in the ELO 3D reconstruction, the top surface again indicates a significant amount of missing material.

### 3.3. Simulation

The lower section of Figure 2 shows the 3D reconstruction obtained from simulation. For part A, the simulation predicts excess material on the side surfaces. For part B, the side surfaces of the lower half are close to the CAD reference with a slight tendency to excess material while for the side surfaces of the upper half a large amount of excess material is calculated. The top surface of both parts indicates missing material due to the consideration of volumetric thermal shrinkage in the numerical data.

## 4. Discussion

The purpose of the investigation was to assess whether in situ ELO imaging is capable of predicting the geometrical and dimensional accuracy of the manufactured parts and to find limitations of the approach which have to be considered for process monitoring applications. Compared to a previous investigation [35] the current evaluation included the complete 3D volume of the parts instead of separately evaluating single 2D layers. Simulation was performed to explain important effects and to validate its capabilities for optimizing PBF-EB scan strategies.

### 4.1. Dimensional Accuracy

The general shape and dimensions of the parts are depicted well by all three methods. Due to thermal shrinkage, all three geometries show missing material on the top side of the parts. The amount of this missing material for ELO and simulation is very similar to the XCT data of the as-built part which indicates that the theoretical thermal shrinkage included into ELO and simulation data was sufficiently accurate. In contrast to the missing material on the top surface, excess material can be found on almost all side surfaces. This observation seems to contradict thermal shrinkage but it may be explained with the scanning strategy. Thermal diffusion and the finite diameter of the electron beam lead to a melt pool with finite dimensions. However, in the current investigation, shape and size of the melt pool were not considered in the design of the beam scanning strategy during melting. Instead, the beam direction during hatching was only reversed when the center of the electron beam reached the contour of the area to be molten, leading to melting and solidification beyond that target contour. The oversize of the part generated by this effect exceeded the volume loss due to thermal shrinkage, resulting in a remaining amount of excess material. Depending on the desired application of a part, these inaccuracies might be problematic and thus be should be corrected. The missing material on the top side of the parts could be simply avoided by slightly scaling up the model according to the expected thermal shrinkage. The compensation of excess material found on the side surfaces is more difficult since the expansion of the melt pool strongly depends on various material properties and process variables, as well as the geometry of parts. Additionally, as shown in a previous investigation [43], the shape and size of the melt pool is also important to obtain stable melting conditions which also is necessary for achieving geometrical accuracy. Thermodynamic simulations may support the development of improved melting strategies, especially for complex-shaped parts with non-steady melt pool properties. This is also shown in the current investigation where simulation of the melt pool extension predicts the occurrence of excess material.

Nevertheless, there are also some geometrical characteristics of the as-built parts which are not depicted accurately by 3D reconstruction from in situ ELO imaging. First of all, the as-built downskin surfaces measured by XCT exhibit a strong edge loss which can not be seen in the ELO images. The effect leads to local deviations between ELO and XCT of up to 500 μm. Most obviously it can be observed at the bottom edges (above the support structure) of both samples. According to the ELO images which were captured directly after melting, the corresponding part cross-sections then had quite accurate dimensions. This proofs that the processes leading to the loss of the edges must have taken place during melting and solidification of subsequent layers. A similar observation was made by Croset et al. [34] for the evaluation of in situ NIR images. Vo et al. [44] proposed an explanation based on the decreasing magnitude of thermal shrinkage during cooling of consecutive overhang layers which is constrained by the growing stiffness of the underlying structure. Ghaoui et al. [16] performed a thermo-mechanical simulation to validate this model and obtained a good agreement with experimental data. The thermo-mechanical origin of the observed effect is also indirectly confirmed by the simulation performed in the current investigation. As the underlying numerical model only includes thermodynamics and not the thermo-mechanical behavior of the system, the edge loss cannot be found in these results.

**Figure 3:**
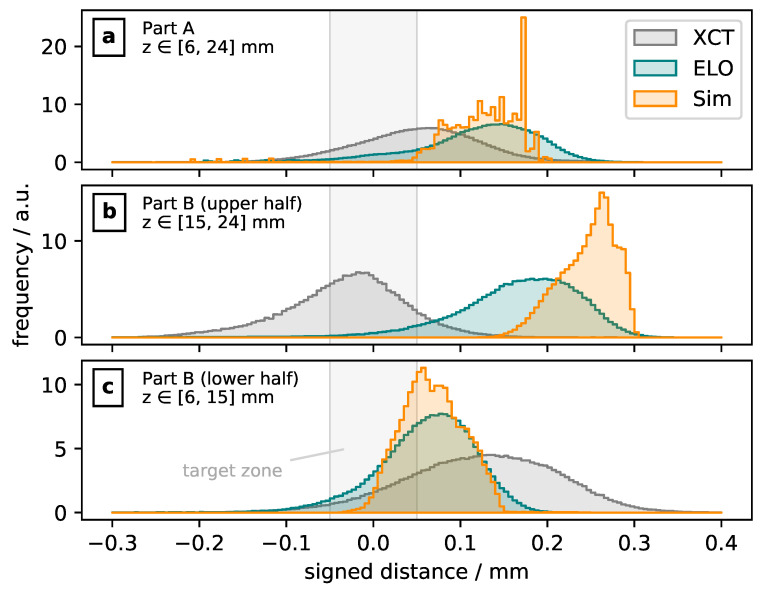
Signed distance histograms of (**a**) part A, (**b**) upper section of part B and (**c**) lower section of part B for XCT, ELO and simulation data.

However, a closer investigation into the accuracy of the side surfaces indicates that additional effects must be involved. A more detailed comparison shows that the amount of excess material predicted by ELO imaging does not perfectly fit the as-built geometry of the part. For part A, the side surfaces of the ELO model show more excess material than the XCT data of the as-built part. This is also displayed in the signed distance histogram of Figure 3a where the peak of the ELO data is clearly shifted by about +90 μm from the XCT peak. This slight overestimation of excess material by ELO imaging was already found for the 2D analysis of molten layers in a previous investigation [35]. One possible reason stated there was the limitation of ELO imaging to surface information. For pure titanium and an acceleration voltage of 60 kV, the model proposed by Kanaya and Okayama [45] estimates an maximum electron penetration depth of around 17 μm [45]. As this is significantly smaller than the melt pool depth, it may be concluded that the BSE information about the material state mainly originates from the upper region of the solidified layer. Thus, it should be noted that the virtual stacking of the ELO images and the subsequent reconstruction does not deliver a real three-dimensional representation of the manufactured part. Instead, the information in z-direction is only an attribute of the two-dimensional ELO images. Therefore, to be more accurate, the reconstructed volume should be perceived as a 2.5D projection of the ELO data. In the previous investigation the analysis was performed on a layer-wise level which involved some uncertainty in selecting the optimum XCT slices for comparison with corresponding ELO images [35]. It was assumed that, due to the curved shape of the melt pool, the molten cross-section of the XCT slice had a high probability of being smaller than its ELO counterpart which always depicts the maximum xy-extension of the melt pool at the surface of the build area. However, the results of the current 3D investigation contradict this theory because XCT analysis of part A still returns less excess material than ELO imaging, even when the complete XCT surface is taken into account.

Another source for the overestimation of excess material might be found in the processing of the ELO data. As explained above, the applied marching cubes algorithm requires a global threshold value as input parameter to extract the surface of the material volume. The selection of this threshold determines which gray level values are considered to represent molten material and thus strongly influences the position of the material-background interface. The choice of the ISO-50% value was supposed to be a neutral approximation but was otherwise more or less arbitrary. A sensitivity analysis revealed that an ELO threshold of 70% delivers a minimum deviation between ELO and XCT data. Further details on the analysis are given in Appendix A. However, it is unlikely that thresholding is the sole source of the deviation while all other (also physical) explanations may be rejected. Usage of the “optimum” thresholding value returned by the sensitivity study might cover relevant effects and distort the results. This assumption is supported by the results obtained from simulation. As shown in Figure 3a, the signed distance histogram of the simulation shows an excellent agreement with ELO data using the original ISO-50% threshold. Since both ELO imaging and simulation display the extension of the molten cross-sections directly after melting, it may be concluded that the ELO threshold was already set correctly. Consequently, usage of an ELO threshold which returns a minimum deviation to XCT data was rejected and instead the ISO-50% value was kept.

The question persists about the cause for the overestimation of excess material by ELO imaging. A deeper insight is gained by including the results of part B into the discussion. Due to the opposed build-up angles involved with this part, its upper and lower half should first be assessed separately. The upper half with the downskin side surfaces shows quite similar results to part A: the ELO data indicates more excess material than actually present. The histograms in Figure 3b enable a more detailed comparison. The figure shows that the spread between ELO and XCT signed distance distribution is actually larger, with the XCT data showing less and the ELO data indicating more excess material than found for part A. For the XCT data, an effect similar to the edge loss at the bottom side due to the continuous overhang across multiple layers may be assumed. The effect seems to be increased at the lateral edges of the pyramidal structure. The reasons are probably the different thermal conditions at the edges and a weaker mechanical fixation due to the surrounding powder bed. Considering the ELO data, the higher amount of excess material is supposed to be related to a larger extension of the melt pool in the overhang region due to different conditions for thermal diffusion. This hypothesis is confirmed by the simulation results which also return a significantly higher amount of excess material than found for part A. By comparing the histograms in Figure 3b it can be seen that the agreement between ELO and simulation is worse in the overhang region. The reason is supposed to be the limitation of the ELO approach to surface imaging of single layers while the simulation also incorporates the melt pool extension in z-direction and layer remelting effects. Further details are given in the following section where surface roughness will be discussed.

The lower section of part B possesses upskin side surfaces which deliver a completely different result than the surfaces discussed before. The ELO data indicates much less excess material while the XCT data displays a large amount of it (see also Figure 3c). This observation is exactly reversed to the ones made before. It may be concluded that the orientation of the surfaces strongly affects the extension of the melt pool, as well as the deformation during melting of subsequent layers. However, it must be noted that the higher amount of excess material seen in the upper part may partly be contributed to the virtual incorporation of thermal shrinkage and the alignment of ELO data and CAD reference at the bottom side of the geometry. Details on this effect are given in the Appendix B. Nevertheless, the corresponding calculation shows that this only accounts for about 60 μm difference between upper and lower section. The remaining effect is assumed to originate from different thermal diffusivity of the subjacent material at the borders of the respective cross-sections. In the lower upskin region, the subjacent layers are always bigger than the layer to be molten. As the bulk material shows a significantly higher thermal diffusivity than the sintered powder-bed (see Table 1), energy is dissipating faster which leads to a smaller extension of the melt pool. In the upper downskin region, the underlying region at the edges consists of sintered powder leading to a stronger accumulation of heat and thus a larger melt pool volume. As can be seen in Figure 3c, the theory is confirmed by the simulation which again shows an excellent agreement with ELO data in the lower half of the part.

**Figure 4:**
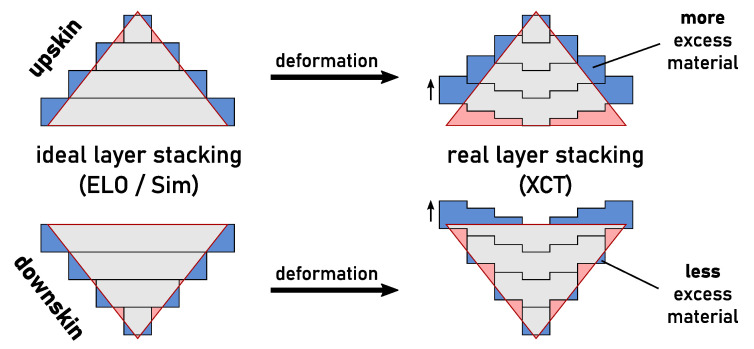
Schematic effect of surface orientation on the accuracy of a part due to an assumed layer deformation induced by thermal stresses.

The difference between upper and lower part as seen in the XCT data might be explained with the deformation of layers induced by thermal stresses. Due to the inhomogeneous temperature distribution during melting and solidification, tensile stresses are created in the upper region of a newly molten layer [10]. The stresses may accumulate during melting of subsequent layers and finally be relieved by plastic deformation, leading to an up-warping of the layer edges. This effect might explain the observed deviations between XCT and ELO/simulation data in the current investigation. Figure 4 illustrates the possible explanation for the effect of surface orientation on the observed deviations. In case of a upskin surface, the material is shifted beyond the target contour of the part where no material is desired. Thus, a increased amount of excess material is found at the sides of the as-built part while material is missing at the bottom edge. In case of a downskin surface, more excess material might be found directly after melting due to the lower thermal diffusivity of the powder bed. However, the stress-induced deformation moves the material upward into the overhang region and therefore less excess material is measured at the side surface of the as-built part. Instead, excess material is found at the edges of the top surface which can also be seen in the XCT data of part B in Figure 2.

Due to the limited number of parts and geometries involved in the current investigation, a full and secure knowledge about the observed effects may not be yet be deduced from the available results. For example, the magnitude of the orientation angle will probably also affect the strength of these upskin and downskin effects. Thus, further experimental investigation is necessary. Nevertheless, the current investigation shows that the combination of in situ ELO imaging and ex-situ measurement of dimensional accuracy enables the distinction between different sources of inaccuracy. This is an important ability for the effective improvement of the parts’ quality.

### 4.2. Side Surface Roughness

**Figure 5:**
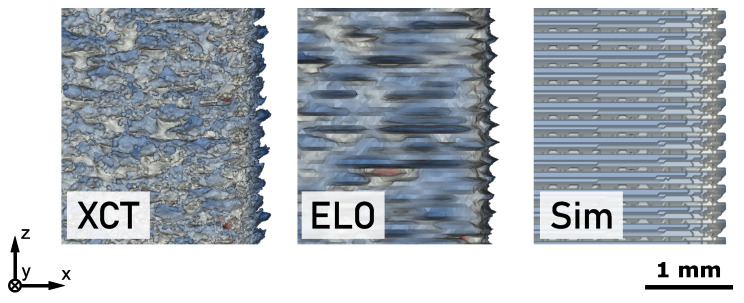
Surface roughness of part A observed in XCT, ELO and simulation data.

The total accuracy of a part consists of the general dimensional accuracy of the bulk volume and a superimposed surface roughness. In powder bed additive manufacturing, the latter is very characteristic along the sides parallel to the build direction and an usually undesired effect of the layer-wise build-up principle. Figure 5 shows the same section of the side surface of part A for the three different 3D models. The XCT measurement of the as-built part displays a typical side surface roughness found for PBF-EB parts. A comparison shows that this roughness may also be found in the 3D model calculated from ELO images. The frequency and also the amplitude of the roughness profile are very similar to those actually found for the as-built part. After it was already shown that ELO imaging can make statements about the in-plane roughness within a layer (i.e., xy-plane) [35], the current investigation reveals that also information on the out-of-plane roughness along the z-axis may be obtained. Considering the restrictions of ELO imaging, i.e., only displaying the surface shortly after melting, this is a very remarkable result. It may be concluded, that the surface roughness must result from a periodic displacement between molten layers, i.e., it mainly is a 2.5D property of the part. The origin for the displacement can be found in the 90° rotation between layers since this creates the observed roughness period length of 200 μm, i.e., four layers. The dynamic formation and variation of the melt pool profile during melting [43] leads to a varying accuracy of the molten layer along its contour. This accuracy is rotated every layer, leading to periodic local displacement even between layers with identical target cross-section. A comparable effect was observed by Croset et al. [34] in NIR images of consecutive layers when the energy input was high [34]. A higher energy input further increases the size of the melt pool and promotes overshooting of the target contour. The associated increase of surface roughness was also measured experimentally [46,47].

This hypothesis is also confirmed by the 3D model derived from the melt pool simulation which can also be found in Figure 5. It also shows the periodic displacement between molten layers which leads to a surface roughness similar to the as-built part. Actually, the roughness predicted by the simulation seems to be even closer to the as-built profile than the one obtained from ELO imaging. While the ELO roughness profile exhibits sharp and thin peaks, the simulation returns thicker extrusions as found by XCT measurement. The reason is that every melting step also affects preceding layers since the melt pool depth exceeds the layer height. ELO imaging only records surface information and thus is not capable of resolving the three-dimensional structure of the molten layer. In contrast, the simulation considers the entire three-dimensional temperature field and hence also is capable of resolving the sub-surface shape of the molten layer. The consideration of melt depth is even more important in downskin regions as found in the upper half of part B. The rather large deviation between ELO and simulation histogram in Figure 3b confirms this assumption. Due to the overhang, the depth of the melt pool generates a significant volume increase which leads to a higher amount of excess material than predicted by ELO data. An aspect which is not included in the current simulation is the effects of hydrodynamics and its interaction with discrete particles in the powder bed. The surface structure returned by the simulation is very clear and periodic because the calculation does not involve any stochastic effects. In contrast, ELO imaging captures the irregular contours of the molten cross-sections which can also be seen in the surface of the 3D reconstruction. The interaction between a dynamic melt pool and the powder particles creates stochastical intrusions and extrusions of the molten contour, leading to missing and excess material of the final part. The associated increase in variance of accuracy might also be visible in Figure 3 where the signed distance distributions of XCT and ELO data always display a higher spread than the corresponding simulation data.

Despite these distinctive features of the different data sets, the main factor for surface roughness in all of them was the local displacement between consecutive layers. This observation is good news for the optimization of surface quality. As the formation and geometry of the melt pool could be controlled using adaptive scanning strategies, the displacement effect may be minimized in the future. Thermodynamic simulations as the one performed in the current investigation could make a substantial contribution in finding these scanning strategies, especially for arbitrary geometries. On the other hand, in situ ELO imaging and analysis can help to immediately validate the optimized strategies during PBF-EB processing which will further enable shortening of development cycles.

**Figure 6:**
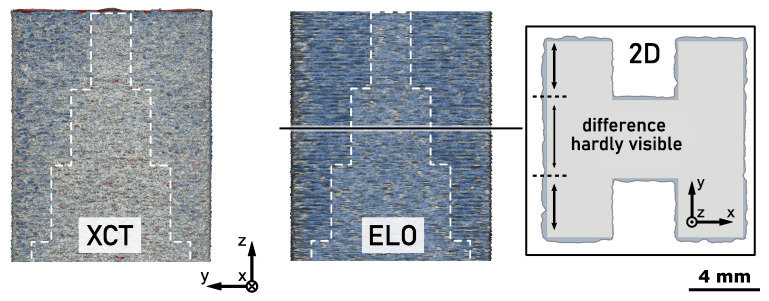
Varying side surface roughness on part A induced by the geometry of the part.

Besides process optimization, ELO imaging is a very promising approach for general monitoring of the PBF-EB process, especially in an industrial environment. This has already been shown for the prediction of internal defects [48,49] and the dimensional accuracy [35]. The current investigation reveals that ELO imaging may also contribute to monitoring of surface roughness. The detail view in Figure 6 shows a special effect found for part A. Its two side surfaces perpendicular to the x-axis exhibit an undesired 2D step pyramid structure. The comparison with the CAD model shows that this step pyramid corresponds to the two-sided staircase in the center section of the part. Those regions where material was step-wise cut out of the cuboid structure display an increased surface roughness on the outer plane. A possible reason for the local deviations might be found in the shorter beam return time when the beam scans across the gap, i.e., every second layer. This might lead to a larger extension of the melt pool but further experiments are necessary to investigate this theory. Figure 6 shows that the step pyramid pattern may also be found in the data derived from ELO imaging. This indicates that the error could have been detected directly during manufacturing of the part. It demonstrates that instead of relying on costly post-process analysis of the as-built parts, ELO imaging enables an early intervention in the PBF-EB process which may spare valuable resources. This specific example also shows a benefit of the 3D analysis of ELO data over a corresponding 2D analysis like performed previously [35]. As shown in Figure 6, using the 2D analysis of single ELO images makes the error hardly visible and thus a detection very unreliable. Only the combination of data across multiple layers gives a clear impression about the extent and source of the inaccuracy. However, it should be noted that the 3D approach is involved with much more computational resources and a more complex analysis of the data.

## 5. Conclusions

The mutual comparison between post-process XCT, in situ ELO and thermodynamic simulation data for two different part geometries enabled an improved distinction between the different effects involved in geometrical and dimensional accuracy of PBF-EB parts. At the same time, the capabilities and limitations of ELO-based predictions for the accuracy of the as-built part were assessed more deeply. The following main conclusions can be drawn from the investigation:

1. In situ ELO imaging can be used to monitor the accuracy of the molten layers and to quantitatively detect deviations from the corresponding reference cross-sections.
2. In situ ELO imaging can be used for making predictions about the out-of-plane surface roughness of the as-built part since it is capable of resolving undesired displacements between consecutive layers.
3. In situ ELO imaging is not capable of predicting all geometrical defects of the as-built PBF-EB parts (e.g., the edge loss effect) because of its limitation to layer-wise imaging of the current surface.

Additionally, the following findings were obtained:

- The combination of in situ ELO imaging and post-process measurement of the as-built part (e.g., via XCT) enables the distinction between the inaccuracy created during melting of a specific layer and the inaccuracies developed during melting of subsequent layers.
- Thermals stresses may induce severe plastic deformation which creates systematic deviations from the reference geometry. Both direction and magnitude of these deviations strongly depend on the characteristics of the involved geometric features (upskin or downskin surface, overhang, edge, etc.). The observed effects might be explained with an up-warping of layers induced by melting of subsequent layers.
- Thermodynamic simulation of the layer-wise melting process and the solidifying volume was demonstrated to be in excellent agreement with experimental in situ data.
- The thermodynamic simulation delivered additional sub-surface information about the molten layers which is important for the full assessment of accuracy and surface roughness, especially for downskin surfaces. In the future, the simulation might be used as a viable tool to optimize scanning strategies in advance in order to improve both accuracy and surface roughness.

In total, the novel combination of post-process XCT measurement, in situ ELO imaging and thermodynamic simulation gave new and quantitative insights into the accuracy obtained by PBF-EB manufacturing. Further investigations should be performed in the future to deepen the understanding of the processes involved and to develop effective countermeasures for improved performance of the manufactured parts.

## Figures and Tables

**Table 1:** Material properties for Ti-6Al-4V [39,40] and simulation parameters.

Property	Unit	Value
Thermal diffusivity solid	m^2^/s	$9\times 10^{-6}$
Thermal diffusivity powder	m^2^/s	$9\times 10^{-7}$
Density	kg/m^3^	4122
Specific heat	J/(kg K)	670
Absorption coefficient		0.85
Liquidus temperature	K	1928
Preheat temperature	K	1023
Beam diameter	μm	400
Lattice spacing	μm	25
Time step	μs	1

**Figure A1:**
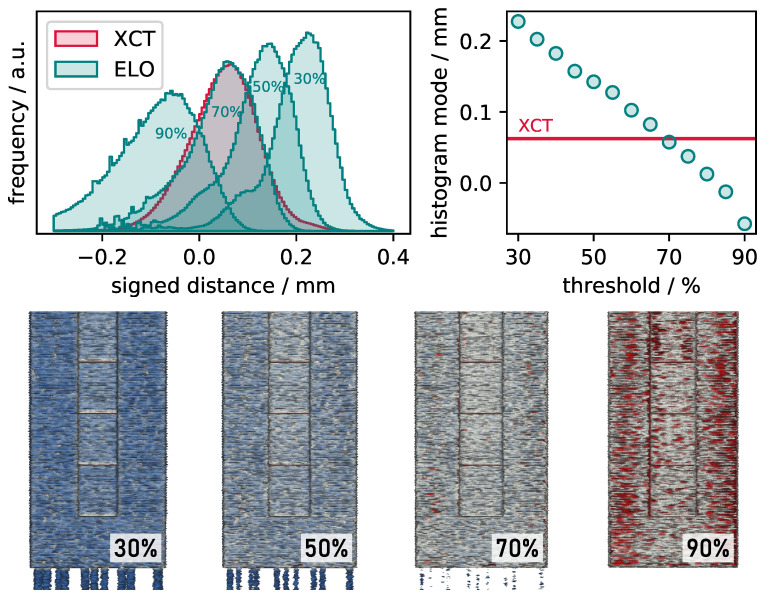
Signed distance analysis of part A for different ELO thresholds.

**Figure A2:**
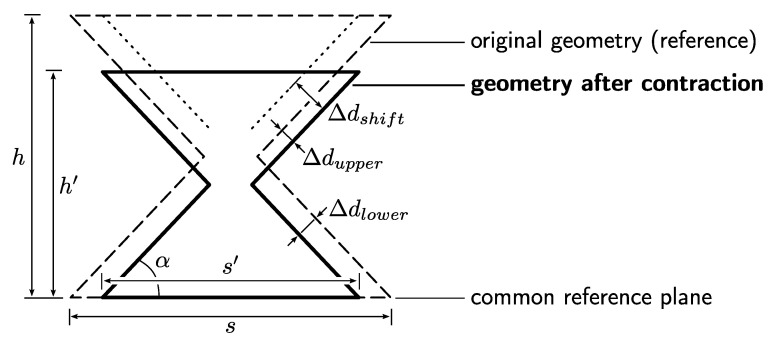
Effect of data registration on the calculated accuracy of the geometry of part B. The alignment of the objects to the bottom side creates an additional shift.

## Data Availability

Data supporting the findings of this study will be available upon reasonable request from the corresponding author.

## Appendix A. Sensitivity Analysis on ELO Thresholding

The marching cubes algorithm used for 3D reconstruction of the parts’ surface from ELO images requires a threshold value that indicates the interface between molten material and powder bed. As explained above, this threshold value was derived from the bimodal distribution of the image gray level values. The center between the material peak and the background peak (i.e., ISO-50% threshold) was supposed to be a neutral choice but is otherwise arbitrary. To investigate the influence of the chosen threshold on the succeeding evaluations, a sensitivity analysis was conducted for part A. The geometry was reconstructed using different threshold values and then compared to the CAD geometry as described above. The lower part of Figure A1 depicts the analyzed 3D geometries for the 30%, 50%, 70% and 90% threshold. As expected, a small threshold value returns a larger amount of excess material (blue) while a big threshold leads to missing material (red). The upper left part of Figure A1 shows the signed distance histograms for the four threshold values. It demonstrates that not only the location but also the spread of the distribution is changing in dependency of the chosen threshold value. For comparison, the histogram corresponding to the XCT analysis of the as-built part is also given. It can be seen that the best agreement is found with the 70% threshold value. For a more detailed analysis, the upper right diagram in Figure A1 gives the mode of the histogram for more ELO threshold values. It confirms that a similar mode as found for XCT data is obtained with an ELO threshold close to 70%. However, usage of this threshold for comparison between ELO and XCT data is not recommended. Further details about this assessment are given in the main part of this article.

## Appendix B. Offset Shift Due to Data Registration

When comparing a geometry to its reference, the data registration, i.e., the transformation into the same coordinate system, strongly affects the obtained result. In particular, this applies for complex geometries with varying cross-section. Different approaches for data registration are possible and the most suitable one should be selected based on the desired aim of the comparison.

In the current investigation, the problem mainly arises due to the thermal shrinkage of the parts which creates a relevant dimensional deviation from the CAD reference model. Due to the simple and symmetric layer cross-section shapes, the registration within the xy-plane was obtained by shifting the respective centroids onto the same position. The registration in z-direction was performed by setting the bottom side of the geometries as common reference plane. This approach was assessed as most reasonable since then the calculated accuracy of a layer is not affected by the number of succeeding layers. However, this choice has some implications on the apparent accuracy. This mainly affects the top surfaces of the parts which indicate a higher amount of missing material. However, also the effect on non-upright side surfaces of the parts must be considered.

In the current investigation, this is the case for part B which possess upskin and downskin surfaces. Figure A2 schematically displays the effect for this geometry. It shows the original geometry with a bottom side length *s* and a height *h*, as well as a shrunken geometry with the dimensions $s^{\prime }$ and $h^{\prime }$. The uniform thermal contraction may be described using the (negative) thermal strain ε as given by Equation (A1). The dihedral angle α is not affected by the contraction. If both geometries were aligned at the narrow center position, the shrunken geometry would indicate missing material for both the upskin and the downskin surface. However, the actual alignment at the bottom side introduces an additional shift $\Delta d_{shift}$ between the upskin and the downskin deviation. In the upskin region of the lower section, the shift leads to a higher amount of missing material with a final distance $\Delta d_{lower}$ to the reference model. In the downskin region of the upper section, the shift not only decreases the apparent amount of missing material but even artificially creates excess material with a final distance $\Delta d_{upper}$ to the reference model. The distances $\Delta d_{shift}$, $\Delta d_{lower}$ and $\Delta d_{upper}$ may be calculated using the Equations (A2)–(A4). The equations show that the strength of the effect is determined by the actual geometry and thermal contraction. Inserting the values of part B (s=10 mm, h=20 mm, $\alpha =67.4{}^{{}^{\circ}}$) and the approximated shrinkage ε=0.77 [35] delivers $\Delta d_{shift}=59\;\mu m$. The theoretical distances to the reference geometry are $\Delta d_{lower}=35\;\mu m$ and $\Delta d_{upper}=24\;\mu m$.

(A1) $$\varepsilon =\frac{s-s^{\prime }}{s}=\frac{h-h^{\prime }}{h}$$

(A2) $$\Delta d_{shift}=\varepsilon h\cos\alpha$$

(A3) $$\Delta d_{lower}=\frac{\varepsilon s}{2}\sin\alpha$$

(A4) $$\Delta d_{upper}=\Delta d_{shift}-\Delta d_{lower}$$
